# Divergent features of ERβ isoforms in triple negative breast cancer: progress and implications for further research

**DOI:** 10.3389/fcell.2023.1240386

**Published:** 2023-10-23

**Authors:** Shunchao Yan, Jinpeng Wang, Hong Chen, Duo Zhang, Murshid Imam

**Affiliations:** Department of Oncology, Shengjing Hospital of China Medical University, Shenyang, China

**Keywords:** ERβ isoforms, triple negative breast cancer, prognostic effect, binding affinity, mechanism, further research directions

## Abstract

Estrogen receptor β (ERβ) was discovered more than 20 years ago. However, the extent and role of ERβ expression in breast cancer remain controversial, especially in the context of triple-negative breast cancer (TNBC). ERβ exists as multiple isoforms, and a series of studies has revealed an inconsistent role of ERβ isoforms in TNBC. Our recent results demonstrated contrasting functions of ERβ1 and ERβ2/β5 in TNBC. Additional research should be conducted to explore the functions of individual ERβ isoforms and develop targeted drugs according to the relevant mechanisms. Consequently, a systematic review of ERβ isoforms is necessary. In this review, we overview the structure of ERβ isoforms and detail what is known about the function of ERβ isoforms in normal mammary tissue and breast cancer. Moreover, this review highlights the divergent features of ERβ isoforms in TNBC. This review also provides insights into the implications of targeting ERβ isoforms for clinical treatment. In conclusion, this review provides a framework delineating the roles and mechanisms of different ERβ isoforms in TNBC and sheds light on future directions for basic and clinical research.

## 1 Introduction

**FIGURE 1:**
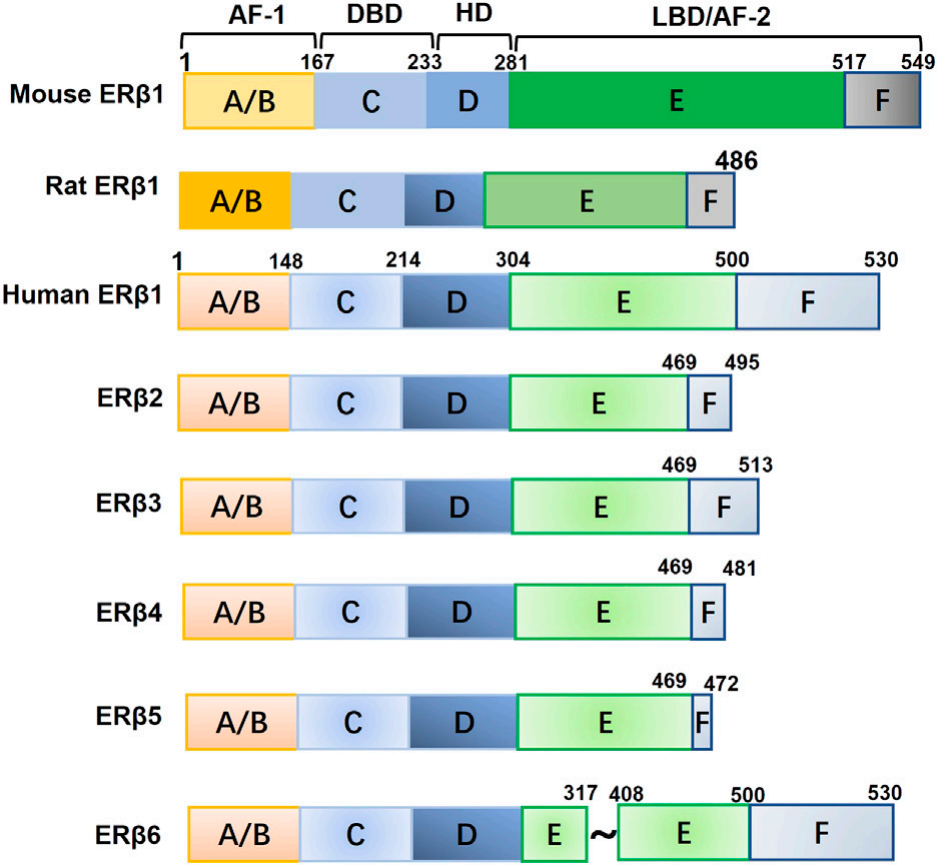
The structure of mouse, rat, and human ERβ1 and other human ERβ isoforms. Abbreviations: AF-1, activation function 1; HD, hinge domain; DBD, DNA-binding domain; LBD, ligand-binding domain; AF-2, activation function 2.

Estrogens are essential for the growth, differentiation, and development of the mammary gland. They are also factors that can promote breast cancer and contribute to its etiology. The physiological and pathological effects of estrogens are primarily conveyed through binding with their receptors. The first estrogen receptor (ER) was identified in 1962 and is now called ERα (Jensen, 1962). In 1996, Kuiper and colleagues (Kuiper et al., 1996) found a novel ER in the rat ovary and prostate, termed ERβ. ERβ is encoded by the ESR2 gene, which is located on chromosome 14q23.2. The full-length human ERβ protein contains 530 amino acids and is encoded by eight exons (Kuiper et al., 1997). ERβ contains five distinct functional domains for ligand binding, nuclear localization, and coactivator/corepressor binding (Enmark et al., 1997). The A/B domain, which is encoded by exon 1, is located at the N-terminus and contains the ligand-independent activation function 1 (AF1). The C and D regions are encoded by exons 2, 3, and 4 and contain the DNA-binding domain (DBD) for nuclear localization and the hinge domain (HD), respectively. The E/F region, encoded by exons 4-8, is located at the C-terminus and contains the ligand-dependent activation function 2 (AF2) and ligand-binding domain (LBD). ERβ2, ERβ3, ERβ4, and ERβ5 are naturally truncated isoforms of ERβ1 that differ after the first 469 amino acids as a result of alternative splicing of the last coding exon (exon 8) (Leung et al., 2006). ERβ6 is an isoform that is truncated in the middle of the protein (Tonetti et al., 2003; Ishii et al., 2021). The functions of ERβ isoforms may diverge given differences in their three-dimensional structures and abilities to bind to ligands and other molecules. In addition, the function of ERβ may differ among humans, mice, and rats because different lengths and ligand binging affinities have been observed for these orthologs (Petersen et al., 1998; O’Brien et al., 1999; Iwamoto et al., 2003; Donoghue et al., 2017; Schröder et al., 2022) (Figure 1). The full-length wild-type ERβ1 isoform is typically referred to as ERβ, unless otherwise stated.

## 2 ERβ isoforms in normal breast tissue and TNBC

### 2.1 Expression of ERβ isoforms in normal breast tissue and their effects on breast cancer development

ERβ1 is the predominant ER in normal breast tissue (Leygue et al., 1998; Speirs et al., 2002), although it is also expressed in the normal tissues of other organs and in endothelial cells, myoepithelial cells, and surrounding stromal cells (Förster et al., 2002; Speirs et al., 2002). An *in vivo* study showed that the mammary gland develops and functions normally in ERβ1-knockout mice, indicating that ERβ1 may not be essential for mammary gland development and function (Krege et al., 1998; Förster et al., 2002). ERα is known to mediate cell proliferation during mammary development. However, some studies have demonstrated that ERβ1 suppresses cell growth, promotes differentiation during mammary development, and decreases the risk of ERa-positive breast cancer (Thomas and Gustafsson, 2011; Dall et al., 2018; Warner et al., 2020).

ERβ1, ERβ2, and ERβ5 have been shown to be expressed in human adult mammary fibroblasts (Palmieri et al., 2004). As lesions progress from being preinvasive to invasive, ERβ1 protein expression decreases in the normal breast (Roger et al., 2001; Shaaban et al., 2003; Skliris et al., 2003). ERβ1 methylation is higher in BC tissues than in normal tissues, resulting in lower levels of ERβ1 mRNA (Gao et al., 2016). It is well known that atypical hyperplasia significantly increases the risk of breast cancer. In one study assessing the expression of ERβ1 using PPG5/10 antibody, levels of ERβ1 protein were lower in atypical lobules than in normal lobules. Further, higher ERβ1 expression was associated with a two-fold decrease in the risk of breast cancer subsequent to atypical hyperplasia (*p* = 0.04), demonstrating the protective effect of ERβ1 against the cancerous process (Hieken et al., 2015). Esslimani-Sahla et al. examined the expression of ERβ2 protein in normal breast and ductal carcinoma *in situ* (DCIS). They found that ERβ2 expression was higher in DCIS than in normal tissue, demonstrating that this may be an early and critical event in the process of carcinogenesis (Esslimani-Sahla et al., 2005). ERβ3 is typically expressed in the testis and prostate tissue (Aschim et al., 2004) but has not been detected in a breast cancer cell line or tumor sample (Tong et al., 2002). ERβ4 has been reported to support the transformation of non-cancerous cells to tumorspheres and to play a role in anchorage-independent growth of mammary epithelial cells (Faria et al., 2018). ERβ5 is abundantly expressed in breast tissue (Moore et al., 1998; Poola et al., 2005b) but may be unable to support tumorigenesis (Faria et al., 2018).

### 2.2 Expression of ERβ isoforms in TNBC

The positive rate and expression level of ERβ1 mRNA is very low in clinical breast cancer samples, according to our analysis of The Cancer Genome Atlas (TCGA) data and others’ reports (Andersson et al., 2017; Yan et al., 2021). The majority of *in vitro* and *in vivo* studies have focused on the mRNA expression of endogenous ERβ isoforms and studied the effect of ERβ isoforms after knockdown or exogenous overexpression of ERβ isoforms. According to our recent study and others’ reports, ERβ2 and ERβ5 are the predominant isoforms in breast cancer and are widely expressed in different molecular types of breast cancer (Andersson et al., 2017; Yan et al., 2021). ERβ3 is not detectable in breast cancer samples or cell lines (Tong et al., 2002). Our TCGA analysis indicated that ERβ4 mRNA was detectable in invasive breast cancer but not in a breast cancer cell line (Yan et al., 2021).

Western blotting (WB) is extensively used for the qualitative detection of proteins. Immunohistochemistry (IHC) and immunofluorescence (IF) are widely used to assay the expression and location of protein in cells and tissue. Sensitivity and specificity of the primary antibody are the key factors that determine the WB, IHC, and IF results. There are several commercially available ERβ antibodies; however, IHC and IF assays of clinical samples and breast cancer cell lines still produce inconsistent results as to the actual expression of ERβ isoforms in breast cancer. These conflicting results are due to the different sensitivities and specificities of ERβ antibodies. In general, ERβ antibodies can be divided into two categories based on the ERβ domain targeted. In theory, antibodies that target the N-terminal or middle domain of ERβ should recognize all ERβ isoforms. Antibodies that target the C-terminus of specific isoforms should recognize only those specific isoforms. There are no consistent results concerning the efficiency and specificity of ERβ antibodies, although these topics have been discussed in several reviews (Pavao and Traish, 2001; Andersson et al., 2017; Nelson et al., 2017). While some authors have claimed that MDA-MB-231 cells are ERβ1 positive (Austin et al., 2018), others have reported that they are ERβ1 negative (Alexandrova et al., 2020a). The mainstream view, based on recent results, is that endogenous expression of ERβ1 protein is negative in cell lines. The available cell lines do not express sufficient endogenous ERβ1 protein to explore its effect in wild type cells (Alexandrova et al., 2020a). These limitations of ERβ antibodies continue to restrict progress in ERβ isoform research. The development of more specific and sensitive antibodies for different isoforms is fundamental to promoting ERβ isoform research.

## 3 The ligand binding affinity of ERβ isoforms

E2 is the natural ligand of ERα and ERβ. There are several synthetic agonists similar to E2 that exhibit better binding affinity with ERβ. The molecular structure of full-length ERβ has 12 helices. Helices 11 and 12 provide a pocket for the ligand and agonist (Pike et al., 1999; Aschim et al., 2004). A molecular modeling study showed that the LBD domain of ERβ1 is very similar to that of ERα and can form a complete helix 11 and 12 when bound to a ligand. ERβ2 may form a complete helix 11 but only a truncated helix 12 because of its shortened C-terminus, which results in a decreased binding surface for the coregulator (Leung et al., 2006). ERβ4 and β5 can only form helix 11 and completely lack helix 12. Ogawa et al. first assayed the binding affinity of E2 for human ERβ isoforms vs. ERα after overexpression of ERα or ERβ in COS-7 cells. The radiolabeled E2 assay results showed that ERβ1 could bind with E2, but its binding affinity was less than that of ERα (Ogawa et al., 1998a; Ogawa et al., 1998b). As shown in Table 1, human ERβ2 exhibited weak binding affinity with E2 (Ogawa et al., 1998b). Poola et al. assayed the binding affinity of E2 with ERβ4 and ERβ5 in COS-7 cells after transfection with either isoform. The ^3^H-labeled estrogen assay indicated that ERβ4 and ERβ5 could not bind to E2 (Poola et al., 2005a). However, Leung et al. found that both ERβ4 and ERβ5 could bind with estrogen using recombinant protein extracted from yeast, but both had lower binding affinity than ERβ1 (Leung et al., 2006). In addition, mouse and rat ERβ2 exhibits weak binding affinity with ligands (Petersen et al., 1998; Zhao et al., 2005). Hence, ERβ1 binds with ligands, but the ligand binding affinity of other human ERβ isoforms is quite low or undetectable. The development of specific ligands for ERβ1 is important for ERβ1 research and potential clinical treatment. Other ERβ isoforms may act mainly in a ligand-independent manner because of their weak ability or incapability to bind to ligands.

**TABLE 1:** Characteristics of studies reporting the binding affinity of ERβ isoforms with ligands.

Species	Isoform	Ligand	Method	Cell model	Results	References
Human	ERβcx/ERβ2	E2, radiolabeled	Ligand binding analysis	COS-7 cells with overexpression of ERβ2	ERβ2 showed little binding affinity with ligand	Ogawa et al. (1998b)
Human	ERβ4,5	^3^H-labeled estrogen	Ligand binding analysis	COS-7 cells with overexpression of ERβ isoforms	ERβ4 and ERβ5 did not bind to E2	Poola et al. (2005a)
Human	ERβ1,2,4,5	^3^H-labeled estrogen	Ligand binding analysis	HEK293 cells transiently expressing ERβ isoforms	ERβ1 could bind with E2. ERβ2 did not bind to E2. ERβ4 and ERβ5 could bind with E2	Leung et al. (2006)
Mouse	ERβ1,2	^3^H-labeled estrogen	Ligand binding analysis	HEK293 cells transiently expressing ERβ isoforms	ERβ1 could bind with E2. The binding affinity of estradiol was 14-fold higher for ERβ1 than for ERβ2	Zhao, et al. (2005)
Rat	ERβ2	Tritiated estradiol	Ligand binding analysis	293T cell transfected with Rat ERβ2	ERβ2 showed weak binding affinity for estradiol	Petersen et al. (1998)

## 4 Prognostic role of ERβ isoforms in TNBC

Most early studies reported the role of ERβ in TNBC without discriminating between isoforms. The majority of the clinical data on ERβ isoforms was analyzed based on the results of IHC or RT-PCR of ERβ isoforms. The prognostic effect of ERβ protein isoforms is unclear given the lack of a specific and sensitive antibody (Nelson et al., 2017; Hawse et al., 2020); furthermore, there is still no standard cutoff value for determining the positivity of cells for ERβ isoforms. Some studies have explored the mRNA expression of ERβ isoforms, but the mRNA expression pattern did not completely overlap with the expression of the functional protein. In addition, ERβ isoforms are also expressed in stromal cells (Green et al., 2008), which may influence the results of RNA analysis. The dominant perception is that ERβ1 promotes survival in ERα-negative BC (Nakopoulou et al., 2004; Rosin et al., 2014; Sun et al., 2018; Shalabi et al., 2021), although some studies have indicated that ERβ1 expression is not associated with outcomes of patients with TNBC (Heitz et al., 2019; Takano et al., 2023). High ERβ2 mRNA and nuclear protein expression have been reported associated with worse outcomes in ERα-negative breast cancer, especially TNBC (Chantzi et al., 2013; Yan et al., 2021; Choi et al., 2022). The prognostic effect of ERβ5 has not been well studied in ERα-negative breast cancer. In our previous study, we analyzed TCGA clinical data and the mRNA expression of ERβ isoforms, observing that high expression of ERβ5 was not associated with disease-free survival or overall survival in patients with TNBC (Yan et al., 2021). In addition to clinical prognostic studies, studies focused on the underlying mechanisms of ERβ isoforms may indirectly shed light on the prognostic role of ERβ isoforms.

## 5 Mechanism underlying the roles of ERβ isoforms in TNBC progression

### 5.1 Mechanism underlying the role of ERβ1 in TNBC progression

**FIGURE 2:**
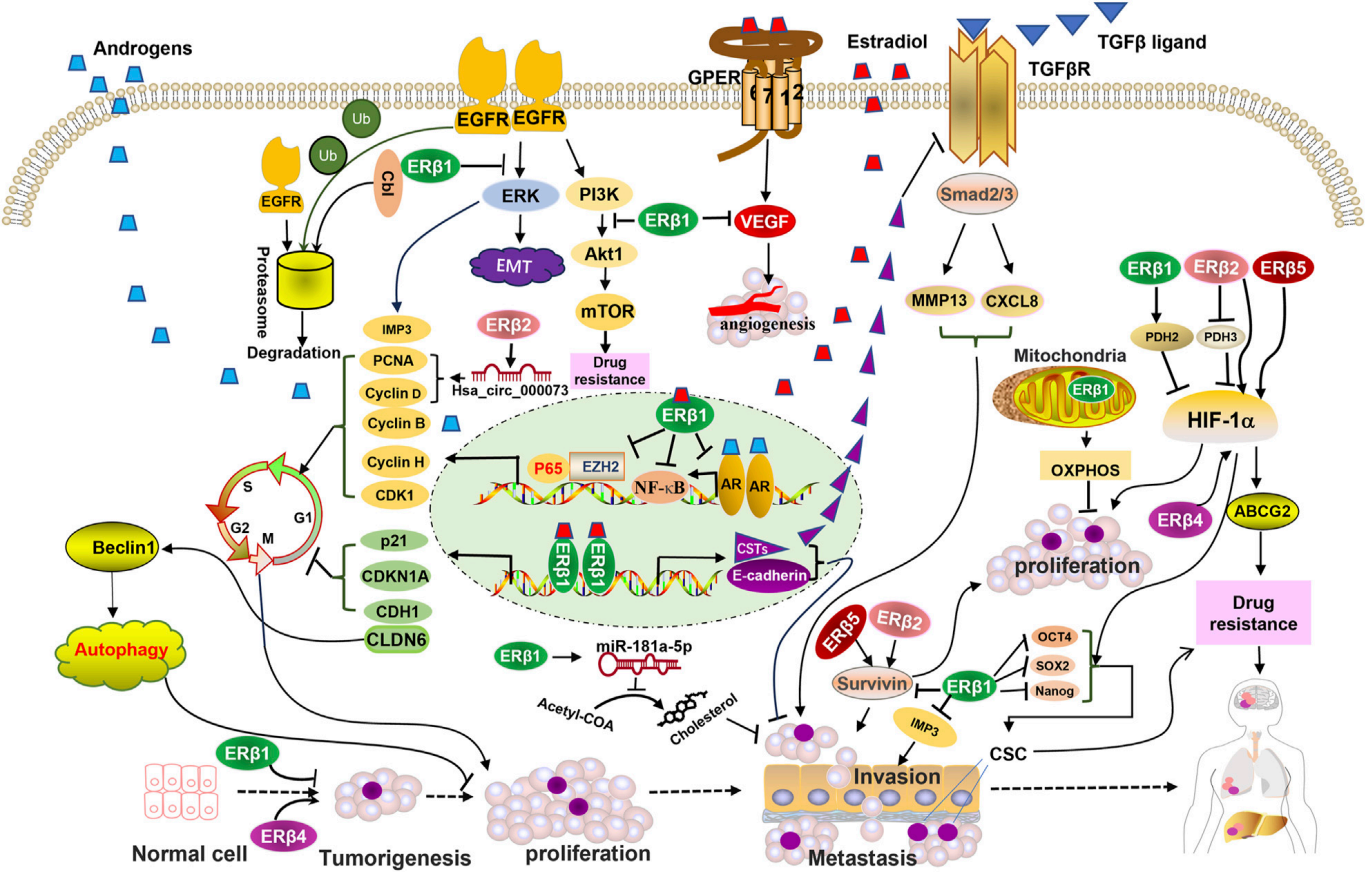
Schematic representation of ERβ isoforms-activated pathways and their interactions with membrane receptors, nuclear receptors, transcription factors, and mitochondrial pathway proteins that are involved in the development and progression of triple-negative breast cancer (TNBC). ERβ1 mediates EGFR degradation and suppresses the activation of downstream EGFR signaling. ERβ1 promotes the transcription of genes that inhibit the cell cycle and the TGF-β signaling pathway, induces autophagy, and suppresses cholesterol biosynthesis. ERβ1 suppresses the transcription of genes that promote the cell cycle. Mitochondrial ERβ1 enhances mitochondrial transcription and activates the oxidative phosphorylation (OXPHOS) system to inhibit TNBC cell growth. ERβ2, 4, and 5 upregulate the HIF-1a pathway and enhance proliferation and drug resistance, opposing the function of ERβ1. ERβ4 upregulates cancer stem cell (CSC) markers, which are inhibited by ERβ1. Abbreviations: CSTs, cystatins; OXPHOS, oxidative phosphorylation; Ub, ubiquitin.

ERβ1 is predominantly located in the nucleus. Nuclear ERβ1 forms complexes with other nuclear receptors and potential nuclear protein partners and binds to the enhancer region of various transcription factors to regulate gene expression and the cell cycle (Charn et al., 2010; Zhao et al., 2010). Cytoplasmic ERβ1 may directly regulate the activity of membrane receptors, downstream pathways, and cholesterol biosynthesis in a ligand-independent manner. Mitochondrial ERβ1 is involved in the regulation of mitochondrial function (Figure 2). Most functions of ERβ1 are not carried out through ligand binding, although they may be dependent on the DNA binding domain. Recent research has indicated that disrupting ERβ1’s direct contact with DNA eliminates its capacity to control the expression of rapid response genes and leaves it unable to control TNBC cell growth (Aspros et al., 2023).

#### 5.1.1 ERβ1 regulates the activation of membrane receptors and downstream pathways

EGFR is deregulated and acts as an oncogenic factor in TNBC (Martin et al., 2012). ERK1/2 and AKT are downstream signals of EGFR. ERβ1 enhances the association of ubiquitin ligase c-Cbl and EGFR and subsequently induces EGFR degradation, which terminates EGFR-activated ERK and impedes epithelial–mesenchymal transition (EMT) in a ligand-independent manner (Thomas et al., 2012). In addition, ERβ1 directly suppresses the PI3K/AKT/mTOR signaling pathway, which is responsible for sensitizing TNBC to doxorubicin treatment (Lei et al., 2020). Insulin-like growth factor II (IGF-II) mRNA-binding protein 3 (IMP3) enhances the invasion and migration of TNBC (Kim et al., 2018). EGFR induces IMP3 transcription and expression through activation of the ERK pathway. ERβ1 may indirectly inhibit IMP3 expression by repressing EGFR, which suppresses the migration and invasion of TNBC (Samanta et al., 2012). However, Kyriakopoulou et al. have reported that ERβ mediating EGFR induces aggressiveness and stemness of TNBC (Kyriakopoulou et al., 2020; Kyriakopoulou et al., 2022).

G protein-coupled estrogen receptor 1 (GPER1), a member of G protein-coupled receptors (GPCRs), is activated by estradiol, and GPER1 expression is correlated with increasing aggressiveness of TNBC (Girgert et al., 2019; Xu et al., 2022). In a recent study, the anti-invasive effect of ERβ agonists was increased by GPER suppression (Schmitz et al., 2022); however, ERβ1 did not directly regulate the expression of GPER mRNA. In ERa-negative inflammatory BC cells, ERβ1 suppresses cell migration via direct suppression of GPR141 expression (another GPCR) (Thomas et al., 2021). Additionally, increased VEGF expression due to increased GPER expression promotes angiogenesis and cancer progression (De Francesco et al., 2014). ERβ1 re-expression and activation have recently been shown to reduce the expression of the VEGF protein, ultimately inhibiting angiogenesis in TNBC (Salahuddin et al., 2022).

The TGFβ signaling pathway plays a critical role during the progression of TNBC (Welm, 2008; Drabsch and Ten Dijke, 2011). Matrix metalloproteinase 13 (MMP-13) promotes tumor invasion and metastasis by mediating the degradation of the epithelial basement membrane and extracellular matrix (Zhang et al., 2008). The chemokine CXCL8 mediates the progression of breast cancer (Mishra et al., 2021). Downregulation of ERβ1 activates TGFβR, subsequently inducing the transcription of MMP-13 and CXCL8. Cystatins are secreted proteins that inhibit the TGFβ pathway. Reese et al. reported that overexpression or ligand-induced activation of ERβ1 inhibits TNBC invasion and migration by inducing cystatin expression and suppressing the TGFβ pathway (Reese et al., 2018). Our recent study showed that overexpression of ERβ1 suppresses the metastasis and invasion of TNBC cells by upregulating the expression of cystatins in both ligand-dependent and ligand-independent manners, and by increasing E-cadherin transcription in a ligand-dependent manner (Yan et al., 2021). Our *in vivo* results further indicated that ERβ1 suppressed both primary tumor growth and metastasis, which was accompanied by a reduction in EMT markers and breast cancer stem cell markers (Dey et al., 2022).

#### 5.1.2 ERβ1 interacts with nuclear receptors and transcription factors

The androgen receptor (AR), a member of the nuclear receptor superfamily, is a strong driver of proliferation in prostate cancer. ERβ1 exerts a tumor-suppressive effect by negatively regulating the expression and activity of AR in prostate cancer (Chaurasiya et al., 2020). Approximately 10%–43% of patients with TNBC are AR positive (Ogawa et al., 2008; Niemeier et al., 2010). Activation of AR enhances the progression of TNBC. Anti-androgen treatment (AR antagonist) is currently being developed for AR + TNBC but is only beneficial for some specific patients (Gucalp et al., 2013; Bonnefoi et al., 2016). The PI3K/AKT pathway is highly activated in AR + TNBC, which is responsible for anti-androgen resistance (Coussy et al., 2020). ERβ1 suppresses AR-mediated cell proliferation by directly heterodimerizing with AR or indirectly suppressing the PI3K/AKT pathways in a ligand-independent manner, which reverses anti-androgen treatment resistance in AR-positive MDA-MB-453 TNBC cells (Anestis et al., 2019). The migration-suppressing effect of ERβ1 was also reported to be mediated by suppressing ZEB1 in AR + TNBC (Song et al., 2017).

EZH2, a transcription factor, is associated with advanced tumor stage, increased mortality, and can promote TNBC progression (Chien et al., 2018; Gan et al., 2018). EZH2 activates gene expression and functions as a coactivator of oncogenic NFκB/p65 signaling in TNBC. Ligand-activated ERβ1 can suppress TNBC growth by acting as a molecular switch for the oncogenic effect of EZH2 and repurposes EZH2 to impart anti-cancer effects (Aspros et al., 2022). On the other hand, ERβ1 can physically associate with NFκB protein and exert anti-tumor effects by inhibiting NFκB signaling in a ligand-independent manner (Aspros et al., 2019).

CDKN1A, p21, and CDH1, three cell cycle inhibitors, have been reported to be upregulated by E2-induced ERβ1 activation (Shanle et al., 2013). In addition, ERβ1 may act as a tumor suppressor, blocking the cell cycle by downregulating other cell cycle-promoting genes including cyclin H, cyclin B, and CDK1 (Reese et al., 2017). Wild-type p53 is a cell cycle checkpoint protein and may inhibit oncogene-mediated proliferation (Eliyahu et al., 1989; Kuerbitz et al., 1992). p53 is another target of ERβ in TNBC, and the mutant status of p53 determines the effect of ERβ (Bado et al., 2016). The majority of breast cancer cases and cell lines contain p53 mutations. Mutant p53 mediates the survival and promotes the proliferation of breast cancer cells (Lim et al., 2009; Arjonen et al., 2014). ERβ1 has been shown to downregulate p53. In p53-mutated breast cancer, ERβ1 inhibits the proliferative and migratory activity of TNBC cells by suppressing the oncogenic function of mutant p53 (Bado et al., 2016), an effect that may be further enhanced by tamoxifen treatment (Scarpetti et al., 2023). However, ERβ has been reported to enhance proliferation in a wild type p53 cell line (Mukhopadhyay et al., 2019). Song et al. also reported that activation of ERβ1 upregulates CLDN6, which induces beclin1-dependent autophagy in TNBC cells (Song et al., 2019b).

Rapidly proliferating cells require cholesterol for biosynthesis of cell membranes and to support cellular biological function. Hence, the factors that regulate cholesterol metabolism are involved in the progression of breast cancer (González-Ortiz et al., 2021). ERβ1 takes part in the regulation of cholesterol biosynthesis in breast cancer cells. ERβ1 regulates many chromatin remodeling complexes, which suppresses breast cancer progression by repressing cholesterol biosynthesis genes (Alexandrova et al., 2020a). miR-181a-5p is involved in the key signaling pathway of cholesterol biosynthesis. It has been reported that ERβ1 inhibits cholesterol biosynthesis by upregulating miR-181a-5p (Alexandrova et al., 2020b).

#### 5.1.3 ERβ1 regulates mitochondrial function

ERβ1 was first identified in the mitochondria of the human heart and aids in regulating mitochondrial function through a genomic pathway (Yang et al., 2004). In ERa-positive breast cancer, E2 treatment may increase ERβ localization in the mitochondria in a time-and concentration-dependent manner (Chen et al., 2004). Studies have shown that in TNBC cells, glucose-regulated protein 75 (GRP75) mediates the translocation of ERβ1 from the cytoplasm to the mitochondria by directly interacting with ERβ1 (Song et al., 2019a). The function of mitochondrial ERβ1 (mitoERβ1) in TNBC remains controversial. Some clinical studies have shown that mitoERβ1 enhances mitochondrial biogenesis to meet the energy demands of tumor progression (Liao et al., 2015). However, others have reported the opposite results, noting that mitoERβ1 suppresses breast cancer progression by maintaining mitochondrial function. Low expression of mitoERβ1 has been associated with an increased risk of postoperative TNBC recurrence. Overexpression of mitoERβ1 enhances mitochondrial transcription, activating the oxidative phosphorylation (OXPHOS) system to produce ATP and inhibit TNBC cells growth *in vitro*, while impairing tumor growth *in vivo* (Song et al., 2019a). In cell culture and mouse xenograft models, these effects were reversed by the deletion of the C- or N-terminal portions of the mitoERβ1 protein. Further investigation demonstrated that full-length mitoERβ1 expression, via binding to the mtDNA D-loop, promotes transcription of 13 mitochondrial genes, an effect that was not observed in the presence of C- or N-terminally truncated receptor versions (Song et al., 2019a). In addition, a clinical study reported that Bcl-2 expression was lower in ERβ1-positive breast cancer than in ERβ-negative breast cancer (Le Cornet et al., 2020). Bcl-2 may suppress apoptosis by inhibiting the mitochondrial permeability transition.

### 5.2 Mechanisms underlying the roles of ERβ2, ERβ4, and ERβ5 in TNBC progression

The expression of ERβ2 and ERβ5 mRNA is higher than that of other isoforms in TNBC. According to our recent study, ERβ2 and ERβ5 are the predominant isoforms and are present in more than 80% of breast cancers (Yan et al., 2021). ERβ2 and ERβ5 are oncogenic and enhance the aggressiveness of TNBC. Exogenous overexpression of ERβ2 or ERβ5 enhances the proliferation, invasion, and migration of TNBC cells by upregulating survivin expression, whereas their downregulation suppresses TNBC progression (Yan et al., 2021).

Accumulating evidence has demonstrated that circRNA is critical for the initiation and progression of TNBC. Hsa_circ_000073, one type of circRNA, is upregulated in TNBC tissues and is positively corelated with the expression of ERβ2. Further studies have indicated that ERβ2 promotes TNBC cell migration and invasion by upregulating hsa_circ_0000732, which upregulates cyclinD1 and PCNA expression (Chen et al., 2022). In addition, mitochondrial ERβ2 drives antiapoptotic pathways in advanced serous ovarian cancer (Ciucci et al., 2015). The role of mitochondrial ERβ2 in TNBC is not clear, marking a key direction for future research.

While ERβ1 preferentially dimerizes with ERβ4, it influences the malignancy of TNBC cells and regulates stem cell markers such as Nanog, SOX2, and OCT4 in an opposing manner (Bano et al., 2023). ERβ4 has been reported to cause mammosphere formation in the human normal mammary epithelial cell line MCF-10A and enhance mammosphere proliferation in the early stages of tumor progression (Faria et al., 2018).

ERβ1 may exert anti-tumor effects in TNBC by suppressing mutation of p53. ERβ2 has been shown to physically interact with mutant p53, increase transcription of the FOXM1 gene, enhance cell proliferation, and lead to carboplatin resistance in patients with high-grade serous ovarian cancer (Oturkar et al., 2022). However, crosstalk among ERβ2, ERβ4, ERβ5, and p53 has not yet been reported in TNBC, providing another valuable direction for future research.

## 6 ERβ isoforms and drug resistance

Hypoxia promotes cell growth, angiogenesis, migration, and drug resistance by activating HIF-1a, the major regulator of oxygen homeostasis. Endogenous ERβ2 and ERβ5 drive the proliferation of TNBC cells by increasing HIF-1a protein levels and upregulating the HIF-1α pathway (Natarajan et al., 2012; Bialesova et al., 2017). HIF-1α expression and transcription are activated by chemotherapeutic drugs in stem-like TNBC cells. HIF inhibitors reverse paclitaxel or gemcitabine resistance and lead to tumor eradication (Samanta et al., 2014). Overexpression of ERβ4 increases HIF-1α expression and increases resistance to paclitaxel in TNBC (Bano et al., 2023). In contrast, downregulation of ERβ4 sensitizes TNBC to paclitaxel (Faria et al., 2018).

Prolyl-4-hydroxylase 1 (PHD1), PHD2, and PHD3—three HIF inhibitors—serve as oxygen sensors in the HIF pathway, hydroxylating HIF-1a in an oxygen-dependent manner (Kaelin and Ratcliffe, 2008). Impeding the catalytic activity of PHDs may stabilize HIF-1a and activate HIF-1a-mediating transcriptional pathways, which can in turn promote cellular adaptation to hypoxic conditions and the transcription of oncogenic genes, thus leading to tumor progression (Lee et al., 2016). ERβ1 has been reported to destabilize HIF-1α by promoting the expression of prolyl hydroxylase 2 (PHD2), which maintains epithelial differentiation and suppresses migration (Mak et al., 2013). Furthermore, ERβ2 has been shown to contribute to the invasiveness of TNBC cells by repressing the transcription of the PHD3 gene and increasing HIF-1α protein levels (Bialesova et al., 2017).

The human breast cancer resistance protein (BCRP/ABCG2) acts to restrict the absorption and regulate the subcellular distribution of drugs (Natarajan et al., 2012). HIF-1a may upregulate ABCG2, which is involved in resistance to cancer drugs and has been correlated with worse prognosis (Krishnamurthy et al., 2004; Staud and Pavek, 2005; Xiang et al., 2012). Overexpression of ERβ2 and ERβ5 has been shown to contribute to drug resistance by increasing the expression of ABCG2 in a TNBC cell line (Faria et al., 2017). Conversely, knockdown of endogenous ERβ2 or ERβ5 can reverse drug resistance in the context of TNBC (Faria et al., 2017; Faria et al., 2019).

## 7 Implications of targeting ERβ isoforms for clinical treatment

Positivity for the ERβ1 protein is detected in approximately 18% of TNBC tumors when analyzed using IHC involving a PPG5/10 ERβ monoclonal antibody, which target the specific C-terminal domain of ERβ1 (Aspros et al., 2022). Ligand-activated ERβ suppresses the aggressiveness of TNBC *in vitro* (Reese et al., 2018; Yan et al., 2021). *In vivo*, ERβ inhibits the growth of TNBC cells in xenograft models and suppresses the development of metastatic lesions in a ligand-dependent manner (Reese et al., 2018; Dey et al., 2022). Thus, targeting ERβ1 using its ligands represents an attractive approach for treating patients with TNBC expressing ERβ1. Estradiol, a form of estrogen, is the natural ligand of ERβ. An ongoing phase II trial at the Mayo clinic is investigating the efficacy of ERβ1 stimulation via estradiol in patients with ERβ1-positive TNBC with advanced or metastatic disease (NCT03941730) (Leon-Ferre and Goetz, 2023). However, results and updates from this trial have yet to be reported but are eagerly awaited.

Preclinical evidence has demonstrated that the oral ERβ agonist S-equol inhibits the proliferation of TNBC cells. A neoadjuvant study evaluated the anti-tumor effects of S-equol in 39 patients with TNBC, reporting that S-equol treatment exerted anti-proliferative effects based on a decrease in Ki-67. Further RNA-seq data indicated that S-equol treatment resulted in immune activation. Future clinical trials designed to assay the synergistic effect of immune checkpoint inhibitors and immune activating agents such as S-equol are warranted (Lathrop et al., 2020; Lathrop et al., 2021). Although several novel synthesized ERβ-selective agonists have also been examined *in vitro*, no clinical trials have been conducted among patients with TNBC (Lathrop et al., 2020; Datta et al., 2021).

Given that cancers are likely to develop *de novo* or acquired resistance to targeted therapy, several studies have explored the mechanisms underlying resistance to ERβ1-targeted therapy. Such studies have reported that lncRNA XIST expression may induce resistance to ERβ1-targeted therapy (Emch et al., 2022). Thus, cases of TNBC with low or no XIST expression may benefit from treatment with ERβ1 agonists. Strategies designed to suppress XIST expression may re-sensitize the resistant cells to ERβ1 agonists. On the other hand, the oncogenic functions of ERβ2, ERβ4, and ERβ5 highlight the potential for the development and clinical application of specific antagonists or receptor down-regulators in TNBC treatment.

## 8 Conclusion

The exact patterns and functions of ERβ isoform expression remain controversial. TNBC cell lines that exhibit detectable full-length ERβ1 protein levels are not available, perhaps because the in vitro-transferable cell lines are more malignant. Clinical prognostic studies focused on the role of ERβ isoforms have also yielded controversial results, possibly due to a lack of sensitive and/or specific antibodies or inaccurate RT-PCR results. Exploring more reliable and precise tools to distinguish different ERβ isoforms is still an urgent problem to be solved. Most of the recently published data on the role of ERβ isoforms were obtained using transient or inducible induction of ERβ isoforms in cell lines. ERβ isoforms exert different effects on proliferation, invasion, and migration in TNBC cell lines. These ERβ isoforms interact with nuclear factors and several signaling pathways, constituting an intricate network that regulates biological behavior in TNBC. Given the inhibitory effect of ERβ1 on TNBC progression, future studies should focus on developing new, specific ligands for study in clinical trials. As for the preliminary data on the carcinogenic effect of ERβ2, ERβ4, and ERβ5, future research should be directed towards exploring novel specific inhibitors or receptor downregulators. Additional studies are required to identify factors that can engage in crosstalk with ERβ2, ERβ4, and ERβ5 to reveal the exact mechanisms by which these isoforms influence TNBC. These data will in turn aid in the development of a scheme for multi-target treatments based on the relevant molecular mechanism.
